# Supplementary material for: Pairwise frictional profile between particles determines discontinuous shear thickening transition in non-colloidal suspensions
Source: Nat Commun. 2017 May 31;8:15633. doi: 10.1038/ncomms15633 (PMC5499204; doi:10.1038/ncomms15633)
Supplement: Supplementary Information — Supplementary Figures and Supplementary Note 1 [file ncomms15633-s1.pdf]

Supplementary Information

1 Supplementary Figures

Supplementary Figure 1 displays an AFM image of one casted PVC particle at the surface of the substrate.

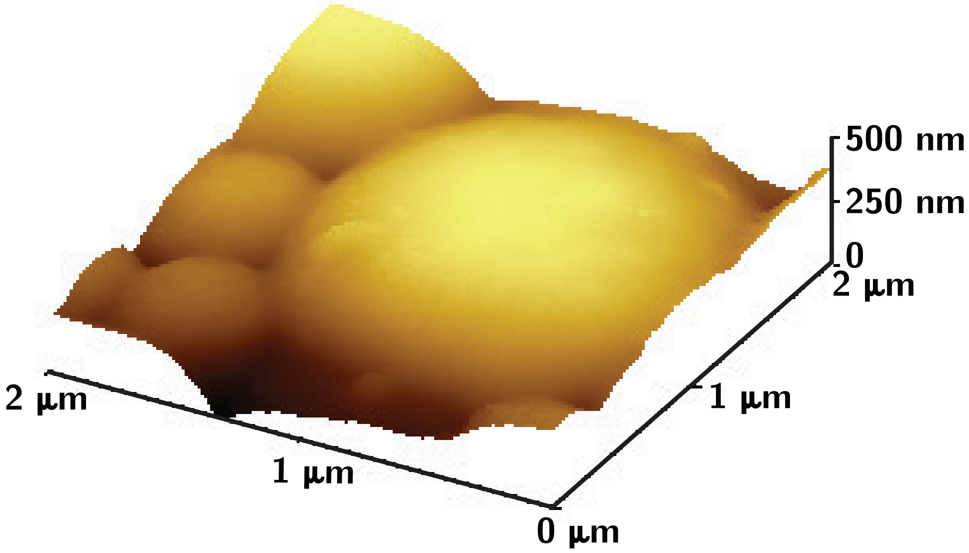

Supplementary Figure 1: **AFM image of one casted PVC particle at the surface of the substrate.** RMS roughness is ≈ 2.2 nm on the upper part of the particle.

Supplementary Figure 2 displays an AFM image of a cornstarch particle glued on a flat silicon substrate. We measured a RMS roughness of 14 nm for the cornstarch particles.

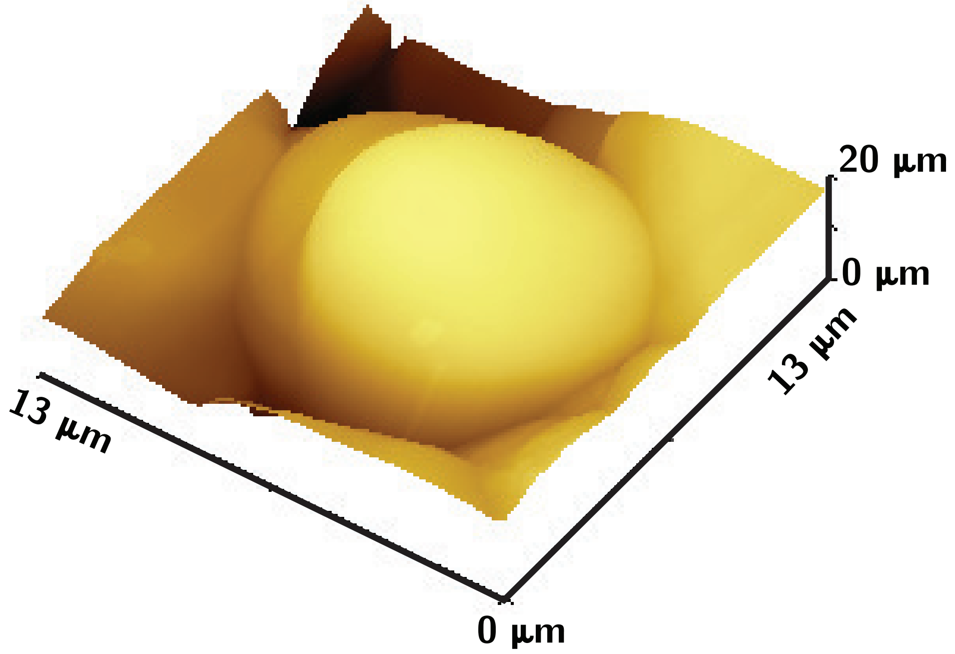

Supplementary Figure 2: **AFM image of one cornstarch particle at the surface of the substrate.** RMS roughness is ≈ 14 nm on the upper part of the particle.

Supplementary Figure 3 displays the rheological curve for PVC and Cornstarch suspensions.

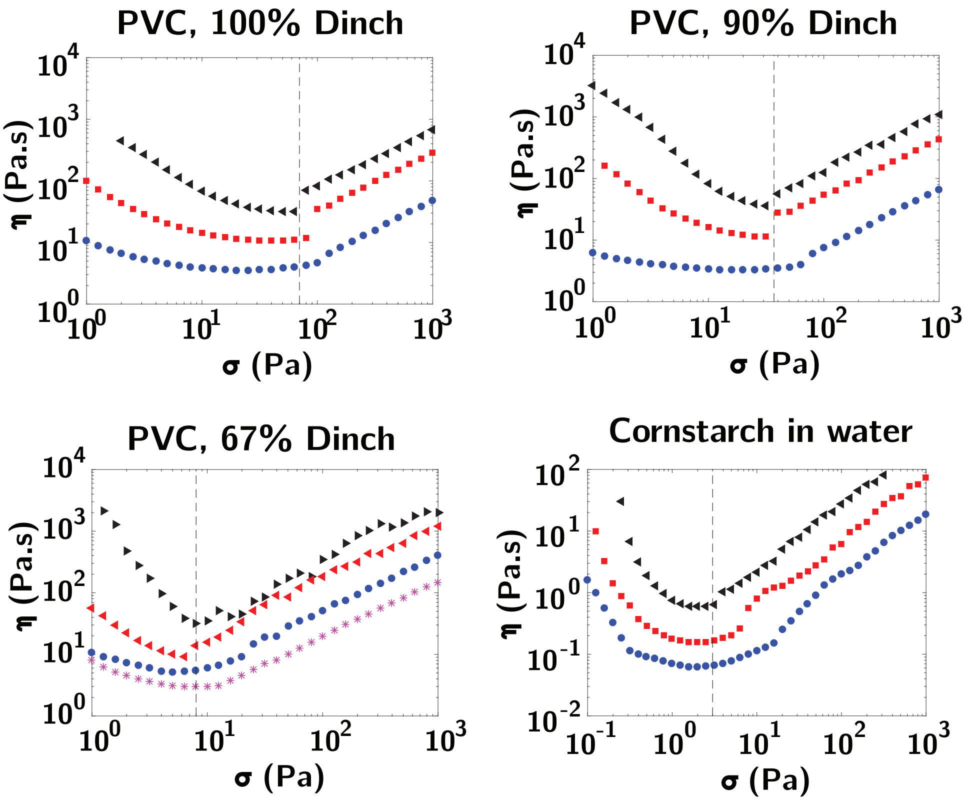

Supplementary Figure 3: Measurements of the viscosity as a function of the shear stress for various solid volume fractions. Top left: PVC, 100% Dinch, from top to bottom the solid fractions correspond to 66%, 64%, 60%. The dotted line corresponds to the shear stress σ_c above which discontinuous shear thickening occurs. Top right: PVC, 90% Dinch; from top to bottom the solid fractions correspond to 66%, 64%, 60%. Bottom left: 67% Dinch, from top to bottom the solid fractions correspond to 63%, 62%, 60% 58%. Bottom right: Cornstarch suspension in water, from top to bottom the solid fractions correspond to 44%, 42%, 40%.

Supplementary Figures 4, 5 and 6 display supplementary data for forces profiles.

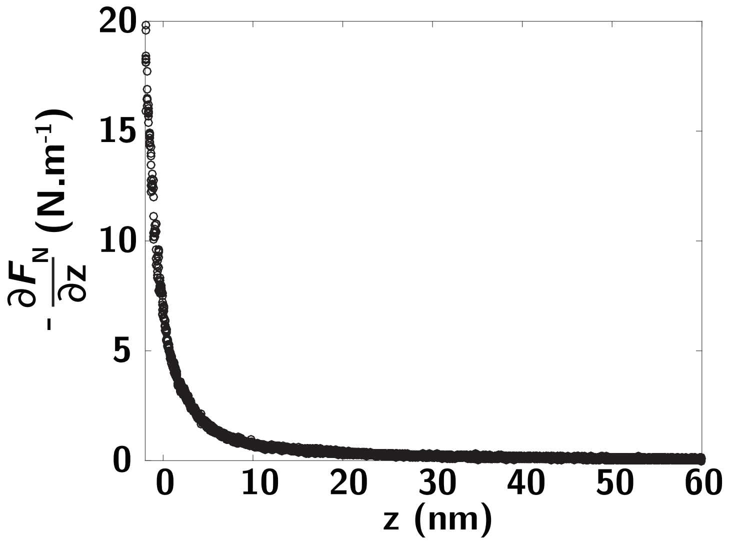

Supplementary Figure 4: **(a)** Normal force gradient $-\partial F_{\mathrm{N}}/\partial z$ for the approach shown in Fig. 2.

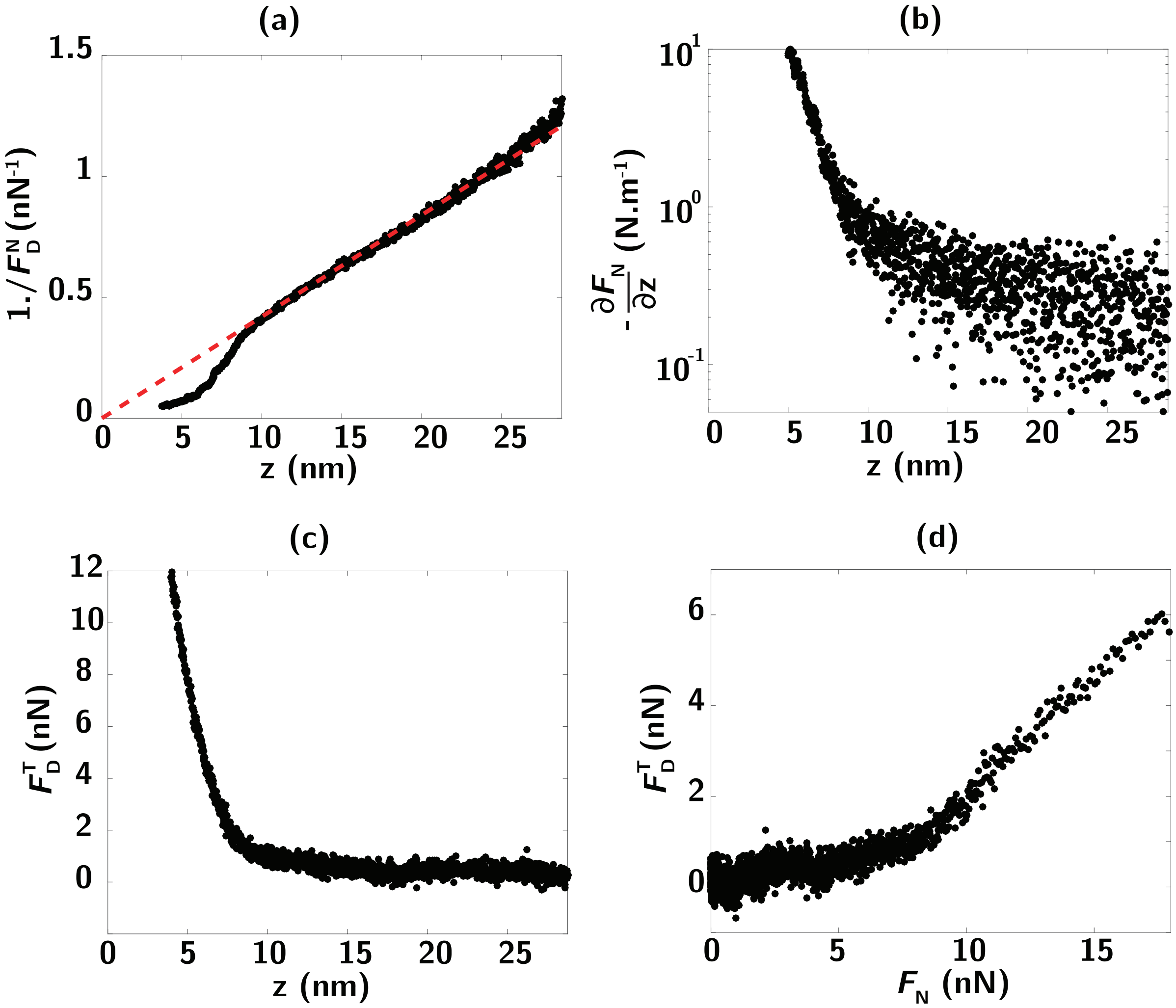

Supplementary Figure 5: **Typical approach in plasticizer (100% Dinch) between two PVC particles. The attached particle is 0.6 µm radius.** Here, contact occurs at ≈ 8 nm before the hydrodynamic zero due to surface asperities on the bottom particle **a)** Inverse normal dissipation. **b)** Normal force gradient **c)** Tangential dissipative force. **d)** Tangential friction force F_D^T versus the normal load F_N.

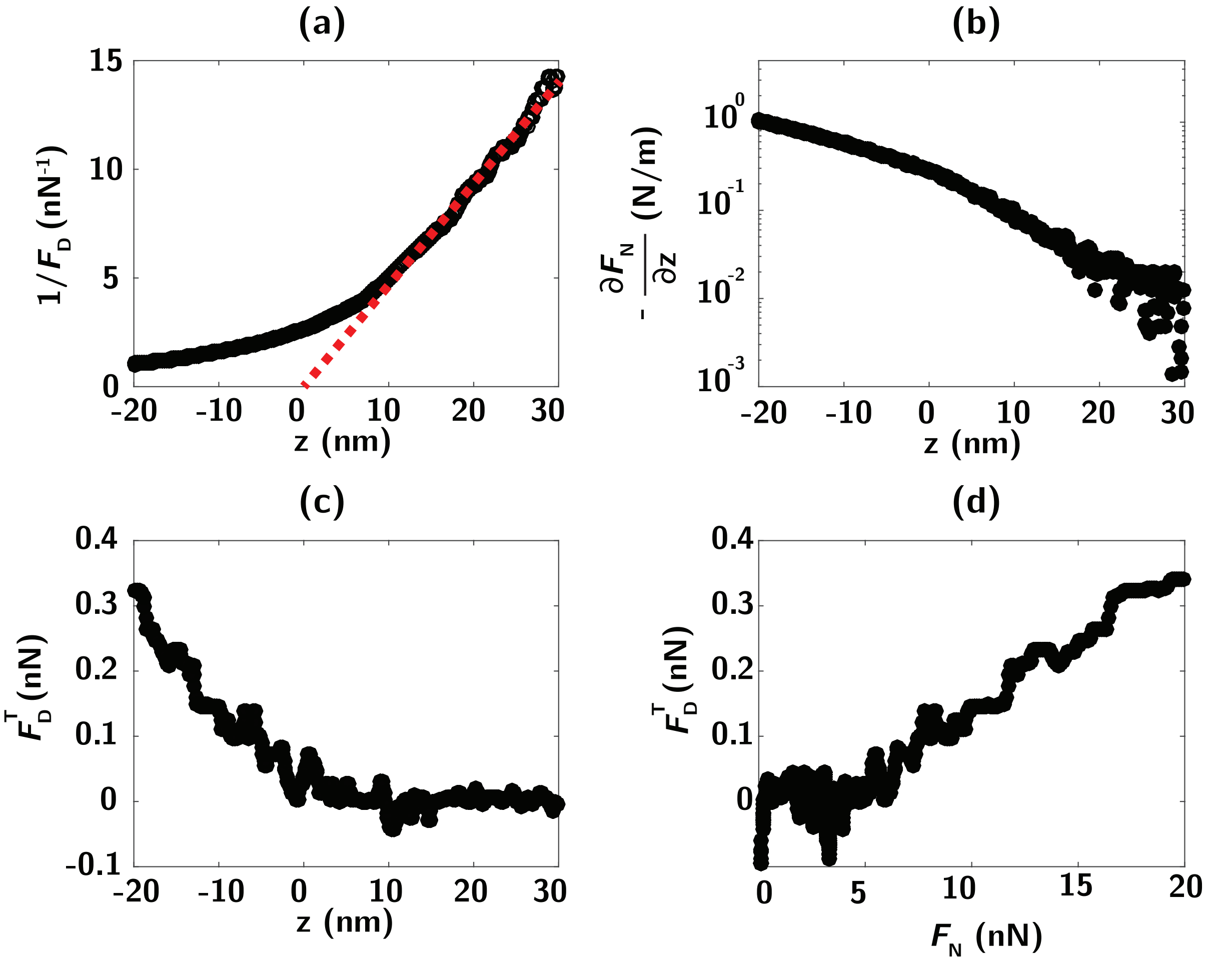

Supplementary Figure 6: **Typical approach curve between two cornstarch particles. The attached particle is 4 µm radius a)** Inverse normal dissipation. **b)** Normal force gradient **c)** Tangential dissipative force. **d)** Tangential friction force F_D^T versus the normal load F_N.

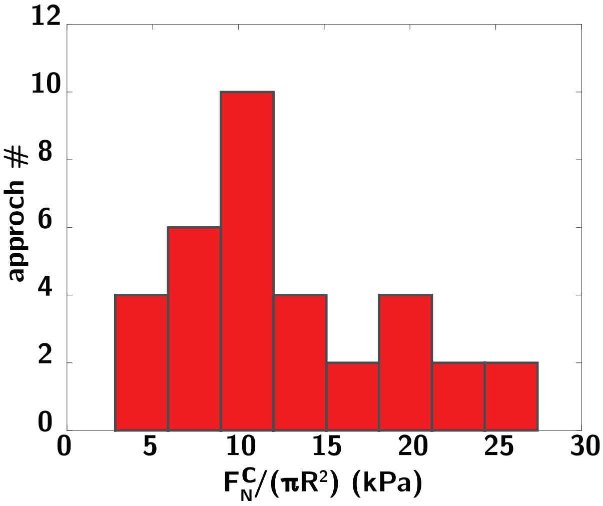

Supplementary Figure 7: **Distribution of normal critical stress for PVC particles in pure Dinch for approaches between 28 distinct PVC particles.**

2 Supplementary Note

Supplementary Note 1: The effective viscosity of the PVC polymer brushes can be measured through a fit of normal dissipation (Reynolds, Eq. (5) of the main text). We first calibrate normal dissipation by approaching the casted particle substrate in a mineral oil of similar viscosity as the plasticizer, but for which no polymer brushes are created. Doing approaches this time in the plasticizer, we find similar viscosities showing that the polymer brushes do not seem to impeed the flow.
